# Supplementary figures and images for: Comparison between flaming, mowing and tillage weed control in the vineyard: Effects on plant community, diversity and abundance
Source: PLoS One. 2020 Aug 31;15(8):e0238396. doi: 10.1371/journal.pone.0238396 (PMC7458340; doi:10.1371/journal.pone.0238396)

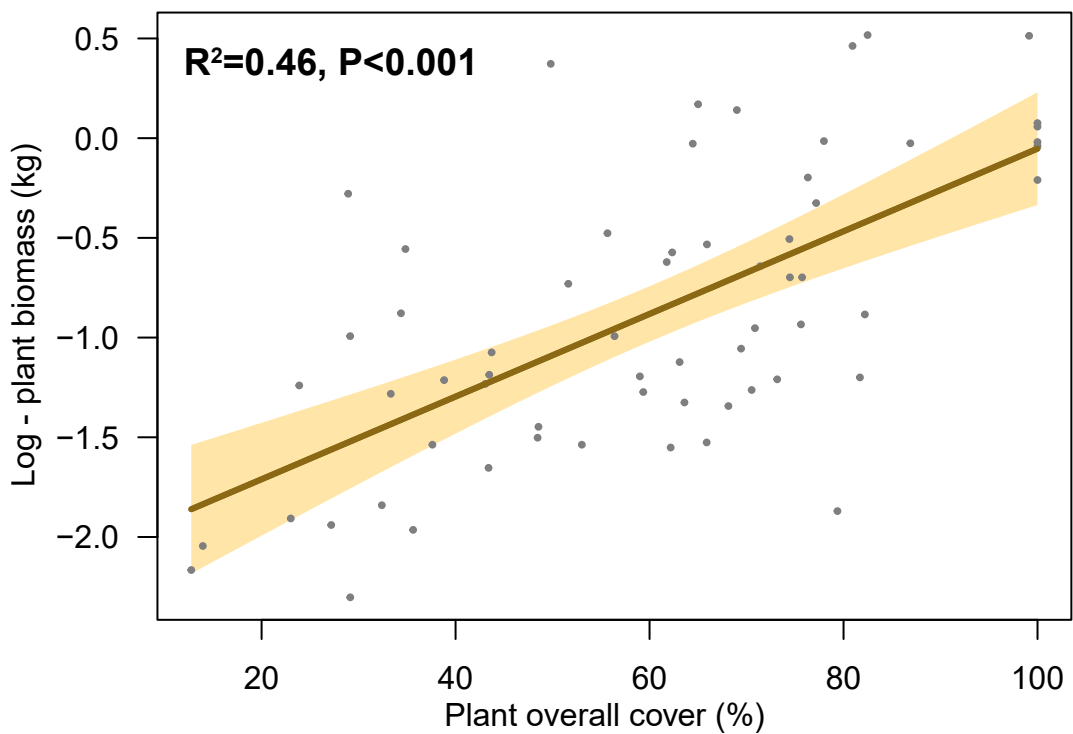

Supplement: S1 Fig — Linear interpolation between measured overall plant cover and measured plant dry matter content at Mid-July. (PDF) [file pone.0238396.s001.pdf]
