# Supplementary material for: Comparison between flaming, mowing and tillage weed control in the vineyard: Effects on plant community, diversity and abundance
Source: PLoS One. 2020 Aug 31;15(8):e0238396. doi: 10.1371/journal.pone.0238396 (PMC7458340; doi:10.1371/journal.pone.0238396)
Supplement: S1 Table — Application dates and forward speeds of the investigated management techniques in the vineyard (intense and gentle flaming, tillage, mowing). (DOCX) [file pone.0238396.s002.docx]

Table S1. Application dates and forward speeds (km/h). Summary of the technical and details of the investigated management techniques in the vineyard (gentle and intense flaming, tillage, mowing).

| **Management technique** |  | **Minimum speed** | **Mean speed** | **Maximum speed** |
| --- | --- | --- | --- | --- |
| Intense flaming |  | 3.7 | 4.1 | 4.6 |
| Gentle flaming |  | 4.3 | 4.8 | 6.0 |
| Tillage (disc cultivator) |  | 4.5 | 4.5 | 4.5 |
| Tillage (weeder blade) |  | 2.6 | 3.2 | 3.7 |
| Mowing |  | 2,2 | 2.4 | 2.7 |
